# Centipede KCNQ Inhibitor SsTx Also Targets K_V_1.3

**DOI:** 10.3390/toxins11020076

**Published:** 2019-02-01

**Authors:** Canwei Du, Jiameng Li, Zicheng Shao, James Mwangi, Runjia Xu, Huiwen Tian, Guoxiang Mo, Ren Lai, Shilong Yang

**Affiliations:** 1 College of Life Sciences, Nanjing Agricultural University, Nanjing 210095, Jiangsu, China; ducw1992@163.com (C.D.); 2017116055@njau.edu.cn (J.L.); 2016116042@njau.edu.cn (Z.S.); 2016116040@njau.edu.cn (R.X.); 2017116051@njau.edu.cn (H.T.); mgx@njau.edu.cn (G.M.); 2 Key Laboratory of Animal Models and Human Disease Mechanisms of Chinese Academy of Sciences/Yunnan Province, Kunming Institute of Zoology, Kunming 650223, Yunnan, China; mwangij1124@yahoo.com; 3 University of Chinese Academy of Sciences, Beijing 100009, China; 4 Sino-African Joint Research Center, Chinese Academy of Science, Wuhan 430074, Hubei, China

**Keywords:** Centipede, SsTx, K_V_7, K_V_1.3, toxin

## Abstract

It was recently discovered that Ssm Spooky Toxin (SsTx) with 53 residues serves as a key killer factor in red-headed centipede’s venom arsenal, due to its potent blockage of the widely expressed KCNQ channels to simultaneously and efficiently disrupt cardiovascular, respiratory, muscular, and nervous systems, suggesting that SsTx is a basic compound for centipedes’ defense and predation. Here, we show that SsTx also inhibits K_V_1.3 channel, which would amplify the broad-spectrum disruptive effect of blocking K_V_7 channels. Interestingly, residue R12 in SsTx extends into the selectivity filter to block K_V_7.4, however, residue K11 in SsTx replaces this ploy when toxin binds on K_V_1.3. Both SsTx and its mutant SsTx_R12A inhibit cytokines production in T cells without affecting the level of K_V_1.3 expression. The results further suggest that SsTx is a key molecule for defense and predation in the centipedes’ venoms and it evolves efficient strategy to disturb multiple physiological targets.

## 1. Introduction

With over 3000 species, centipedes, arthropods of the class Chilopoda are widely distributed in both tropical and subtropical regions. Their first pair of legs have been modified into strong mandibles and venomous fangs called forcipules that play a critical role in defense and predation [1]. Centipede stings occur frequently in areas, such as Hawaii, Brazil, Australia, Taiwan, and Japan [2], evoking an issue of social health care. Centipede toxins are composite mixtures, which are responsible for both local and systemic reactions. Recently, we have identified and characterized several peptide toxins from centipede venom and established they act on ion channels that are associated with pain and inflammation [3,4,5,6,7]. For example, a centipede toxin (RhTx) causes intense local pain by targeting the heat activation machinery of nociceptor TRPV1 (transient receptor potential cation channel subfamily V member 1) channel [4]. We also revealed the molecular basis of cardiovascular, respiratory, muscular, and nervous system’s dysfunction due to centipede bites by the identification of Ssm Spooky Toxin (SsTx). It targets KCNQ channels (voltage-gated potassium channel family 7) to exhibit potent lethal toxicity by causing severe vessels and respiratory disorders or seizures. Mechanically, SsTx binds to K_V_7 channels tightly, due to the fact that residue K13 in SsTx anchors the toxin to the outer pore region of K_V_7 channels and residue R12 in SsTx extends into the selectivity filter to block the channels [8]. Therefore, SsTx shows physiological and pathological importance in understanding the efficiency in prey capture and clinical reports after centipede envenomation. In the present study, we established that SsTx also inhibited K_V_1.3 that have been reported to be an important target for autoimmune diseases [9,10,11]. Through the construction of toxin mutants, we found that SsTx used different side chains to target K_V_1.3, which allowed us to modify them towards specifically targeting K_V_1.3 channel in human T cells. Furthermore, SsTx and its mutant suppressed Tem-effector cells proliferation and cytokine production in T cells.

## 2. Results

### 2.1. SsTx Inhibits K_V_1.3

**Figure 1:**
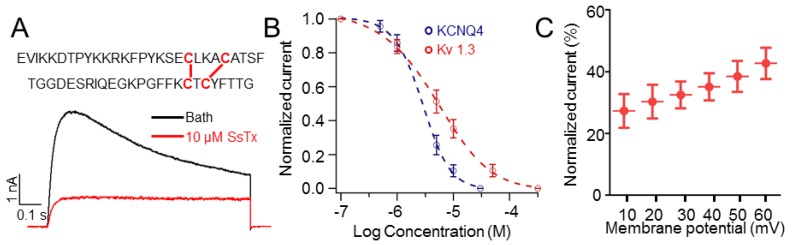
The effect of Ssm Spooky Toxin (SsTx) on K_V_1.3. (**A**) K_V_1.3 currents were inhibited by 10 μM SsTx. The black and red traces represent bath solution and solution containing 10 μM SsTx, respectively. The upper insert shows the sequence of SsTx and the cysteine residues are presented in red color. (**B**) Concentration-response curves displaying the inhibition of SsTx on K_V_7.4 and K_V_1.3. Data were fitted with a Hill equation. The IC_50_ values are 2.80 ± 0.23 μM for K_V_7.4 (*n* = 5 cells) and 5.26 ± 0.56 μM for K_V_1.3 (*n* = 10 cells). (**C**) The relationship between the inhibitory percentage of 10 μM SsTx on K_V_1.3 and the test pulses. The cells were held at −80 mV (*n* = 4–6 cells).

As described in our previous work [8], centipede prey on animals via SsTx inhibiting the K_V_7.4. SsTx consists of 53 amino acids refolded by two intracellular disulfide bonds (Figure 1A). Surprisingly, we found that SsTx toxin also inhibited K_V_1.3. As illustrated in Figure 1A, SsTx potently inhibited K_V_1.3 currents, with an IC_50_ value of 5.26 μM (Figure 1B). As a pore blocker, SsTx inhibited K_V_1.3 channel in a voltage-dependent manner (Figure 1C), similar to the inhibition of K_V_7.4. Taken together, our results, which are consistent with a previous report [12], suggest that the βαββ-type toxins have a higher affinity for K_V_7 and K_V_1.3 channels. The fact that SsTx shows no effect on Slo1 channel implies that the binding surface of the scorpion toxin charybdotoxin may have sophisticatedly evolved to use a different mechanism to inhibit Slo1 channel [13,14,15].

### 2.2. K11 and K13 in SsTx Are Crucial for Inhibiting K_V_1.3

**Figure 2:**
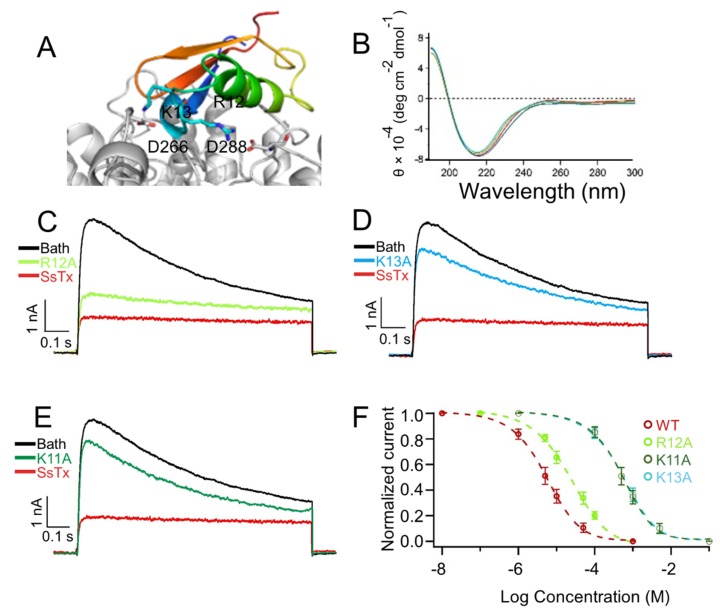
The residues on SsTx altered subtype-selectivity. (**A**) Molecular docking of SsTx onto K_V_7.4. The side chains of R12/K13 in SsTx and D266/D288 in K_V_7.4 are shown. (**B**) CD (circular dichroism) spectra of SsTx and mutants exhibited no significant difference. (**C**–**E**) Representative K_V_1.3 currents were inhibited by 10 μM SsTx_R12A (**C**), SsTx_K13A (**D**) and SsTx_K11A (**E**). (**F**) Dose–response curves displaying the inhibition of SsTx_R12A, SsTx_K13A and SsTx_K11A on K_V_1.3, respectively. The IC_50_ values are 22.23 ± 0.22 μM for SsTx_R12A (*n* = 5 cells), 526.1 ± 0.48 μM for SsTx_K13A (*n* = 5 cells), and 507.0 ± 0.61 μM for SsTx_K11A (*n* = 5 cells), respectively.

Toxins with multiple functions have been widely utilized to probe the structure–function relationship of ion channels [16,17]. Given that SsTx targets both K_V_1.3 and K_V_7 channels, we studied the key residues for their bio-activity on K_V_1.3 and K_V_7 channels. Our previous results demonstrate that there are two direct interactions between SsTx and K_V_7.4: The side chain of K13 on the toxin anchors it to the outer pore region of K_V_7.4, and the side chain of R12 extends into the selectivity filter (Figure 2A). Because blockage of K_V_7 channels is considered to be toxic, such information may direct our functional efforts to modify this native toxin and acquire a more selective K_V_1.3 inhibitor by mutagenesis. To test whether these residues are also critical for SsTx interaction with K_V_1.3 channel, we generated point mutations at these sites. These mutant toxins exhibited typical structural features (Figure 2B). Using alanine substitution, we found that the affinity of mutant SsTx_R12A for K_V_ 1.3 was almost entirely intact (Figure 2C,F). In contrast, the IC_50_ value of SsTx_K13A mutant increased by more than 100-fold for K_V_1.3, suggesting that K13 on SsTx predominantly affects its binding affinity to K_V_1.3 (Figure 2D,F). Next, we wondered whether there was another amino acid that specifically mediates the interaction between SsTx and K_V_1.3. We found that the IC_50_ value of mutant SsTx_K11A increased by more than 100-fold for K_V_1.3 (Figure 2E,F), suggesting the lysine residue at position K11 provides the key side chain that anchors the toxin specifically onto K_V_1.3 rather than K_V_7.4. Therefore, the toxin mutant SsTx_R12A exhibits selectivity on K_V_1.3, which is a likely suitable inhibitor for our future studies.

### 2.3. SsTx and SsTx_R12A Suppress Proliferation of Human T Cells without Affecting the Expression of K_V_1.3

**Figure 3:**
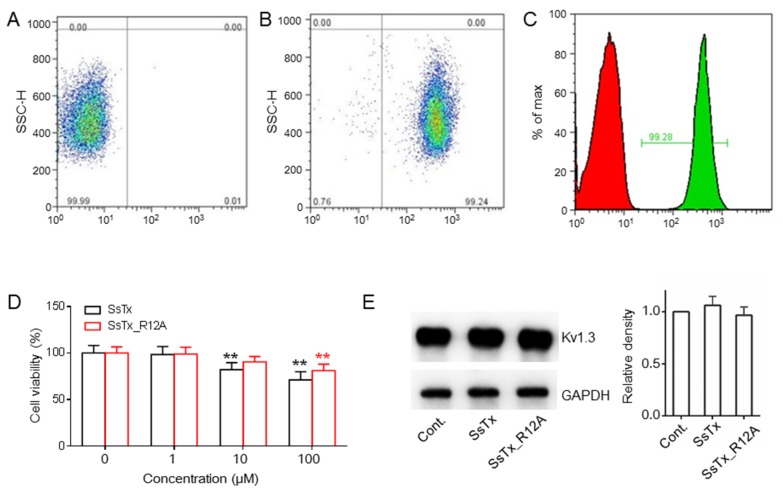
SsTx and SsTx_R12A suppressed proliferation of human T cells without affecting the expression of K_V_1.3. (**A**,**B**) Isolation of human T cells that were incubated with the primary antibody against CD3+ (**B**) compared to saline solution (**A**); SSC-H, side scatter-height. (**C**)The purity of CD3+ T cells was determined by flow cytometry. (**D**) The effect of different concentrations of SsTx_R12A on human CD3+ T cell proliferation compared to the absence of SsTx. ** *p* < 0.01. (**E**) Both SsTx and SsTx_R12A at 100 μM exerted no significant effect on the expression of K_V_1.3 on human CD3+ T cells (left panel). The densities of K_V_1.3 were quantified by band intensity with ImageJ relative to GAPDH (glyceraldehyde-3-phosphate dehydrogenase) (*n* = 3) and GAPDH was used as a loading control (right panel).

The K_V_1.3 channel is expressed abundantly in the immune cell, and it is a target for curing autoimmune diseases. Some molecular compounds [18] and peptides [19] have been used as probes to explore the relationship between K_V_1.3 and autoimmune diseases. For example, SHK-186, the special K_V_1.3 inhibitor, suppresses T cell proliferation without affecting the level of K_V_1.3 expression [20]. Here, we isolated the Tem (Effective Memory T)-effector cells from peripheral blood mononuclear cells (Figure 3A,B). By losing its inhibitory activity to K_V_7.4 but retaining substantial affinity for K_V_1.3, it suggests the mutant SsTx_R12A, after modification, provides a potential therapeutic agent for autoimmune diseases. Additionally, we found both SsTx and SsTx_R12A suppressed Tem-effector cell proliferation in a concentration-dependent manner (Figure 3D) without affecting K_V_1.3 expression even at a concentration of 100 μM (Figure 3E). Taken together, our results demonstrated that SsTx and mutant SsTx_R12A potently blocked K_V_1.3 in human T cells, leading to suppression of cell proliferation.

### 2.4. SsTx and Mutant SsTx_R12A Suppress Cytokine Production in T Cells

**Figure 4:**
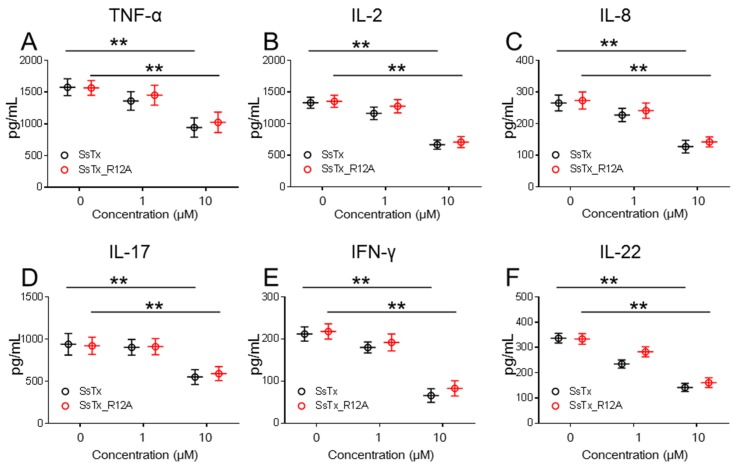
SsTx and SsTx_R12A inhibited the function of human T cells. SsTx and SsTx_R12A inhibited secretion of TNF-α (**A**), IL-2 (**B**), IL-8 (**C**), IL-17 (**D**), IFN-γ (**E**), and IL-22 (**F**) in human CD3+ T cells. ** *p* < 0.01.

Blockage of K_V_1.3 in Tem-effector cells has been reported to suppress the secretion of cytokines, such as TNF-α and IL-2 [21]. Consistent with these observations, both SsTx and mutant SsTx_R12A suppressed the secretion of TNF-α and IL-2 by almost 30% in activated human CD3+ T cells at a concentration of 10 μM (Figure 4A,B). Similarly, SsTx suppressed the production of other cytokines, such as IL-8, IL-17, TFN-γ, and IL-22, in a concentration-dependent manner (Figure 4C–F). Here, we found that SsTx as the first K_V_1.3 inhibitor from centipede venom not only suppressed Tem-effector cells proliferation but also suppressed the production of cytokines in activated human CD3+ T cells. This suggests that the mutant SsTx_R12A, by losing its inhibitory activity against K_V_7.4 but maintaining the activity to K_V_1.3, might be a potential drug for curing autoimmunity diseases.

## 3. Discussion

Until recently, the toxins arsenal of centipedes remained elusive [22], with only a few individual venom components characterized and their function established. Advancements in sequencing and mass spectrometry technologies have given researchers a deeper understanding of the evolutionary process, composition, and possible mechanisms of action of centipede venoms. Although the taxonomical scope of species investigated is still narrow, more studies have established that centipede venom consists of a wide repertoire of toxins possessing novel structural scaffolds. [5,7,23,24]. In our previous studies, among all identified centipede toxins, we established the physiological importance of SsTx in Chinese red-headed centipede venom [8], specifically its involvement in the induction of severe clinical symptoms following centipede envenomation [25,26,27,28].

In this study, we found that the SsTx exhibited potent inhibitory activity on K_V_1.3 (Figure 1). The selective blockage of K_V_1.3 has been established as a viable option for targeting T cell-mediated autoimmune diseases without inducing generalized immune suppression [29,30,31]. Nonetheless, side-effects are often observed in toxin-derived potential therapeutics because of off-targets. Interestingly, R12 in SsTx is crucial for anchoring it onto K_V_7 channels whereas K11 functions in a similar way for binding K_V_1.3 (Figure 2C,F). Taking advantage of the retained affinity to K_V_1.3, we predicted and validated the therapeutic potential of SsTx for autoimmune diseases by suppressing T-cell cytokines production (Figure 4A–F). Our findings on the mutant toxin not only demonstrate a representative method for abolishing toxicity but also present SsTx_R12A and its modified variants as excellent potential therapeutic agents for T cell-mediated autoimmune diseases.

Animal venom is an important bio-resource library, and several peptides, such as SHK-186 [20] from the sea snail, have been used to explore the relationship between ion channels and certain diseases. SsTx plays an important part for the centipede in preying food and defense against predators by inhibiting K_V_7 [8]. Here, we have found SsTx showed a dual function, including its ability to inhibit K_V_1.3. Peptides derived from animal venom exhibit multiple functions, and with the elimination of the side effects of these peptides, they offer excellent prospects for development of drug agents.

## 4. Materials and Methods

### 4.1. Peptides Synthesis and Refolding

Following a 9-fluorenyl methoxycarbonyl/tert-butyl strategy and HOBt/TBTU/NMM coupling method, linear Sstx and its mutant peptides were synthesized on an automatic peptide synthesizer (PerSeptive Biosystems, Boston, MA, USA) as described in our previous work. Peptides refolding were performed by peptides dissolution in a solution containing 10 mM glutathione and 100 mM oxidized glutathione. The solution was adjusted to pH 6.0, and placed at 28 °C for 24 h. A method coupling MODLI-TOF mass spectrometry and HPLC techniques were performed to determine the purity of peptides to be higher than 95%.

### 4.2. Cell Transfection

HEK293T and CHO cells were cultured as described in our previous work [32]. The plasmid DNA (5μL, 1 μg/μL) of K_V_1.3 and K_V_7.4 channel was mixed with lipofectamine at a ratio of 1:1 in 200 μL Opti-MEM for 15–20 min, and the mixture was transferred to the cells plated in 20 mm cell culture dish for 12 h. After 24 h transfections, electrophysiological experiments were carried out at room temperature.

### 4.3. Electrophysiological Recordings

The cells expressing K_V_1.3 and K_V_7.4 were digested and plated on glass coverslips before current recording. The HEKA EPC10 amplifier (HEKA Elektronik, Ludwigshafen, Germany) was used to record currents in whole cells under the control of PATCHMASTER software (HEKA Elektronik, Ludwigshafen, Germany). A thin-wall borosilicate glass (A-M Systems) was used to pull patch pipettes and then the pipettes were fire-polished to 3–4 megohm. For whole-cell recordings, the capacity current was minimized by amplifier circuitry, and the series resistance was compensated by 30% to 65%. For K_V_1.3 and K_V_7.4 channel, intracellular solution contained 140 mM KCl, 10 mM EGTA, 2 mM MgCl_2_, 1 mM CaCl_2_, 10 mM HEPES (pH 7.3, adjusted with KOH), and extracellular solution contained 150 mM NaCl, 0.5 mM CaCl_2_, 5 mM KCl, 1.2 mM MgCl_2_, 10 mM HEPES (pH 7.3, adjusted with NaOH), and the current traces were tested by a 1000-ms depolarizing pulse of 10 mV from a holding voltage of −80 mV. The Hill logistic equation shown below was used to fit the Dose–response curves:

y = 1 − (1 − fmax)/[1 + ([Tx]/IC_50_) n] (1)

where n is an empirical Hill coefficient, and fmax is the fraction of current resistant to inhibition at high toxin (Tx) concentration.

### 4.4. Human T Cells Isolation

Peripheral blood mononuclear cells (PBMCs) were collected from healthy volunteers and T cells were isolated from PBMCs by negative magnetic depletion using biotin-conjugated CD14, CD15, CD16, CD19, CD34, CD56, CD123, and CD235a following the manufacturer’s instructions of Human Pan T Cells Isolation Kit (Miltenyi Biotec, Bergisch Gladbach, Germany). Ficoll–Hypaque density gradient centrifugation was used to separate the PBMCs at 3000 rpm for 15 min under the manufacturer’s protocol. Flow cytometry was carried out to assess the purity of Tem cells to be more than 95% followed by maintaining in RPMI (Roswell Park Memorial Institute) 1640 medium (Thermo, Waltham, MA, USA) containing 10% FBS (fetal bovine serum) (Thermo, Waltham, MA, USA), streptomycin (100 μg/mL) and penicillin (100 U/mL) in 5% CO_2_ at 37 °C for 1 h.

### 4.5. Human T Cells Proliferation and Viability Measurement

Human T cells obtained as described above were seeded into 96-well plate. 20 μL of the test sample dissolved in RPMI 1640 medium was added to the wells followed by 24 h incubation at 37 °C; the same volume of the medium was used as blank control. After incubation, the medium was replaced with MTT (methyl thiazolyl tetrazolim) containing medium (final concentration 5 mg/mL) followed by further incubation for 5 h under the same conditions. Subsequently, the MTT was dissolved with 200 μL dimethyl sulphoxide (DMSO), and then the absorbance of the resulting solution was read at 570 nm. The corresponding cell viability of the treated group was expressed as the percentage viability of the control group.

### 4.6. K_V_1.3 Expression in T Cells

Cellular proteins (20 μL, 1 mg/mL) from differently treated groups were loaded onto a 12% SDS-PAGE gel. After gel electrophoresis at 120 V for 2 h at room temperature, the proteins were transferred onto a PVDF (polyvinylidene fluoride) membrane. The membrane was incubated with a 1:500 dilution of a primary antibody against GAPDH and K_V_1.3 (Abcam) at 2–8 °C for 12 h after blocking in 5% skim milk for 1–2 h at room temperature. The blots were first washed three times with TBST (Tris-Buffer-Solution-Tween) to clean the primary antibody and then incubated with the secondary antibody conjugated by horseradish peroxidase (Abcam) at a 1:1000 dilution. After washing for 3–5 times with TBST, blots development was carried out by chemiluminescence.

### 4.7. Cytokines Secretion by Human CD3+ T Cells

SsTx and its mutant were dissolved in PBS and mixed 1 h before bead stimulation. The dynabeads against CD3+/CD28+ were used to activate the isolated CD3+ T cells for three times at a ratio of 1:1 in 96-well plates. Cells were collected after overnight activation, and cytokines secretion (hTNF-α, hIL-2, hIL-8, hIL-17, hIL-22, and hIFN-γ) was determined by ELISA (R&D, St. paul, MN, USA) under the manufacturer’s instructions.
